# Fetal cardiotocography before and after water aerobics during pregnancy

**DOI:** 10.1186/1742-4755-7-23

**Published:** 2010-08-31

**Authors:** Carla Silveira, Belmiro G Pereira, Jose G Cecatti, Sergio R Cavalcante, Rosa I Pereira

**Affiliations:** 1 Department of Obstetrics and Gynecology, School of Medical Sciences, University of Campinas-UNICAMP, Campinas-SP, Brazil; 2 Department of Anesthesiology, School of Medical Sciences, University of Campinas- UNICAMP, PO Box 6030, 13083-881 Campinas-SP, Brazil

## Abstract

**Objective:**

To evaluate the effect of moderate aerobic physical activity in water on fetal cardiotocography patterns in sedentary pregnant women.

**Method:**

In a non-randomized controlled trial, 133 previously sedentary pregnant women participated in multiple regular sessions of water aerobics in a heated swimming pool. Cardiotocography was performed for 20 minutes before and just after the oriented exercise. Cardiotocography patterns were analyzed pre- and post-exercise according to gestational age groups (24-27, 28-31, 32-35 and 36-40 weeks). Student's t and Wilcoxon, and McNemar tests were used, respectively, to analyze numerical and categorical variables.

**Results:**

No significant variations were found between pre- and post-exercise values of fetal heart rate (FHR), number of fetal body movements (FM) or accelerations (A), FM/A ratio or the presence of decelerations. Variability in FHR was significantly higher following exercise only in pregnancies of 24-27 weeks.

**Conclusions:**

Moderate physical activity in water was not associated with any significant alterations in fetal cardiotocography patterns, which suggests no adverse effect on the fetus.

## Background

In the past, the practice of physical activity by pregnant women has always been surrounded by uncertainties and was considered a taboo. Sedentary approaches were encouraged and women were counseled to stay off their feet as much as possible. In addition, the fear that women could acquire genital infections from immersion in water caused health professionals to discourage this activity [1]. Currently, there is an almost general recognition among health professionals that moderate exercise offers no risks to pregnant women as long as there are no obstetrical complications or preexisting conditions, such as vaginal bleeding, arterial hypertension or cervical incompetence [2].

In 1985, the American College of Obstetricians and Gynecologists (ACOG) issued the opinion that exercise during low-risk pregnancy is safe for the mother and fetus when maternal heart rate does not exceed 140 beats per minute for more than 15 minutes [3]. Recent guidelines issued by the ACOG recommend caution and medical supervision to avoid any possible harmful effects of the practice of physical activity of moderate intensity for 30 minutes or more, preferably every day of the week; however, maximum maternal heart rate for this practice has not been established [2,4]. The recommendations of the Royal College of Obstetricians and Gynaecologists (RCOG) for the practice of physical activity during pregnancy define predetermined arbitrary limits of maternal heart rate for each age-group of patients. Therefore, for women under 20 years of age, a heart rate (HR) between 140 and 155 bpm is recommended; for women 20-29 years of age, HR should remain between 135 and 150 bpm; from 30 to 39 years of age, HR should remain between 130 and 145 bpm; and for women >40 years of age, HR should be between 125 and 140 bpm [5].

The effect of the regular practice of physical exercise during pregnancy on fetal heart activity and its vitality has not yet been studied in depth, although some studies have shown the relationship between maternal physical activity and the physiological responses of increased fetal heart rate [6-8]. Few studies have been carried out in pregnant women in water and many questions remain to be answered on this subject, although no harmful effect of this practice on the fetus has been reported [1].

The growing concern with maternal health and fetal well-being, together with advances in technological development, have encouraged a more detailed evaluation of heart rate and other parameters of fetal homeostasis, with the objective of assuring the safety of practicing aerobic physical activity during pregnancy and as a requirement for the recommendation of its regular practice.

The objective of this study was to evaluate the effect of moderate physical activity in water on pregnancy, particularly the parameters evaluated by antepartum fetal cardiotocography (CTG) prior to and following physical exercise at different gestational ages in the second and third trimesters. Variables comprised: basal fetal heart rate (FHR); fetal body movements (FM); accelerations (A); ratio between fetal movements and accelerations (FM/A ratio); variability in FHR and transitory decelerations.

## Methods

This is a non-randomized, controlled, before/after clinical trial carried out in pregnant women at different gestational ages to compare fetal cardiotocography parameters prior to and following moderate physical exercise in water. Sample size was calculated considering a fetal heart rate prior to (149 ± 6 bpm) and following swimming at 35 weeks of gestation [9] to detect a minimum difference of 4 bpm between the two periods, with alpha and beta errors of 5%, resulting in a minimum sample size of 58 cases per gestational age-group.

The study was carried out at the Department of Gynecology and Obstetrics of the University of Campinas, Brazil. Pregnant women were recruited to the study between March 2004 and September 2006. Inclusion criteria comprised: previously sedentary pregnant women with gestational age ≥ 24 weeks, low-risk pregnancy and a single fetus. Exclusion criteria were: high-risk pregnancy, contraindications to the practice of physical activity, hypertension of any etiology, placenta previa, history of repeat abortions or preterm labor, smoking or regular alcohol consumption, regular use of medications, and participation in any regular program of physical activity. All women recruited to the study provided written authorization from the physician responsible for their prenatal care and signed the study informed consent form prior to enrollment. The study was approved by the Institutional Review Board prior to initiation. The women included in the study were provided with tickets for transportation, as well as the material they required to participate in the water aerobics sessions (swimsuit, cap, towel, bag, sandals, etc.).

The multiple regular sessions of water aerobics were carried out in a covered, heated (28-30°C) pool. Each session lasted 50 minutes and consisted of moderate intensity exercise, following the protocol of the ACOG [10] in which four phases of exercise are recommended for greater safety: warm-up and stretching exercises, aerobic session, resistance exercises and a cool-down period or return to resting condition. The moderate intensity exercise means that the ideal heart rate calculated should be adapted to 60 to 90% of one's age predicted maximum heart rate [11,12]. This was followed using a heart rate counter in a belt placed in the woman's thorax. Sessions were conducted under the professional guidance of a physical education teacher or physiotherapist, and were supervised by a trained nurse, who was also responsible for carrying out the cardiotocography exam. All the sessions took place in middle-morning or middle-afternoon, around two hours apart from breakfast or lunch. Fetal monitoring was carried out using a Sonicaid Team^® ^fetal monitor equipped with a Care Printer. The women were monitored for 20 minutes prior to the entire water aerobics sessions and immediately following the session, in the lateral decubitus position or seated. Women were always checked for fetal body movements while performing monitoring, registering each movement with an event marker linked with the fetal monitor. The exam was recorded on the device, which later printed the FHR register for detailed analysis. The analysis measures fetal heart rate parameters, performs a test that compares these results with criteria defined as normal, and highlights any abnormalities. Besides this electronic assessment of cardiotocography patterns, the exams were independently assessed by two other examiners and the values were considered for results only when a consensus was achieved for each parameter.

A database was built with double data entry of all cases, after which data was cleaned, a consistency check was made, entries were corrected whenever necessary, and statistical analysis was performed. Student's t-test was used for samples related to FHR evaluation. Wilcoxon's non-parametric test was used for samples related to variation in the number of fetal body movements (fetal body movements as perceived and registered by women), A (accelerations - an increase of at least 15 beats during at least 15 seconds) and variability in FHR (a beat-to-beat variation of at least 5 beats). McNemar's test with binomial distribution was used to analyze the FM (fetal body movements)/A ratio (considered normal when at least 2/2 ratio was achieved in 20 minutes) and decelerations. All tests were carried out on measurements recorded prior to and immediately following physical activity.

## Results

A total of 243 pre- and post-immersion examinations were performed at four time-periods during the second and third trimesters of pregnancy (at 24-27, 28-31, 32-35 and 36-40 weeks of gestation) in 133 pregnant women, representing a mean of 1.8 evaluations per woman. The results reported here consist of the variables measured by cardiotocography prior to and immediately following moderate exercise in water (water aerobics). As shown in Table 1, most of the women in the study were between 25 and 34 years of age, with no previous history of abortion, and were pregnant for the first time.

**Table 1:** Characteristics of the women included in the study

*Characteristics*	*n*	*%*
*Maternal Age (years)*		
≤ 19	9	6.8
20 - 24	23	17.3
25 - 29	57	42.9
30 - 34	29	21.8
≥ 35	15	11.3
*History of abortions*		
Yes	16	12.0
No	117	88.0
*Number of pregnancies*		
1	76	57.1
2	32	24.1
3 or ore	25	18.8
*Parity*		
0	80	60.2
1	36	27.1
2	11	8.3
3 or more	6	4.5
**Total**	**133**	**100**

**Figure 1:**
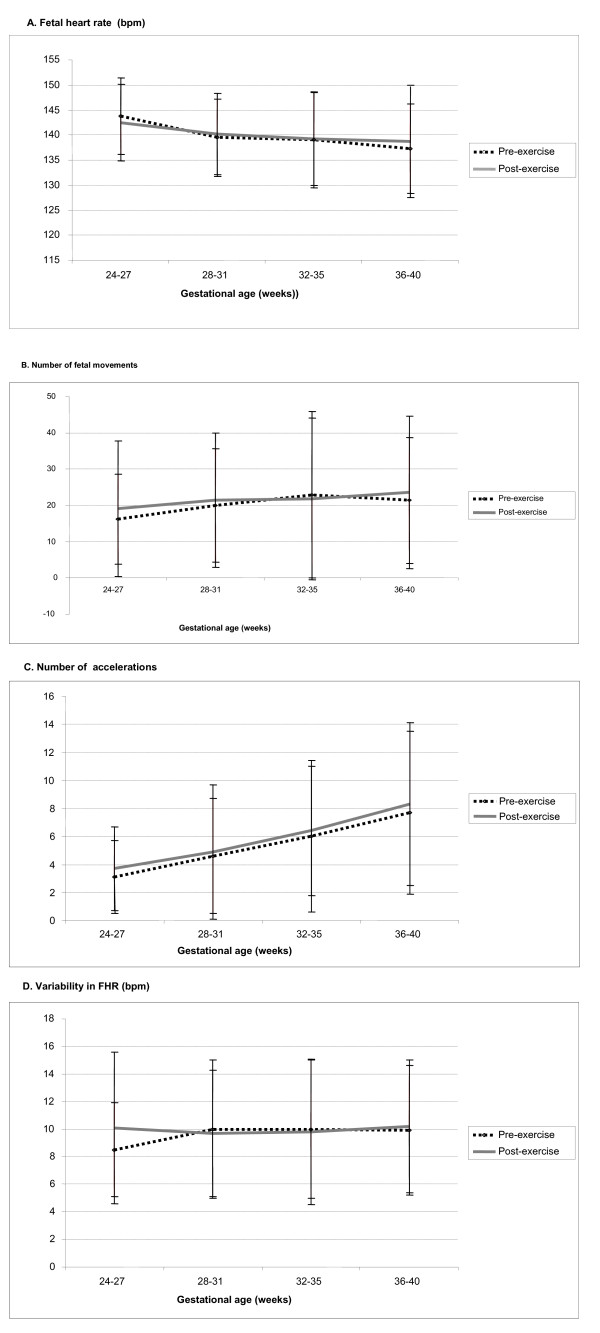
**A. Mean fetal heart rate (± SD)**; **B**. Mean number of fetal movements (± SD); **C**. Mean number of transitory accelerations (± SD); and **D**. Variability in FHR (± SD) prior to and following moderate physical exercise in water, according to gestational age-group.

According to the comparative evaluation of cardiotocography measurements prior to and following water aerobics, no statistically significant variations were registered in basal fetal heart rate between the two time-periods. Likewise, there were no significant differences in the mean number of fetal body movements or accelerations registered within the period of 20 minutes of the exam. Moreover, the variability in FHR in general was not significantly different before and after water aerobics except in the earliest gestational age-group evaluated (24-27 weeks) when FHR was significantly higher following water aerobics (Table 2). Figure 1 illustrates these values.

**Table 2:** Cardiotocography (CTG) recorded variation in basal fetal heart rate (FHR), in the number of fetal movements (FM), in the number of accelerations (A) and in variability of FHR before and after moderate physical exercise in water, according to gestational age (GA).

*GA (weeks)*	*Basal FHR (mean ± SD)*				
			
	*CTG before*	*CTG after*	*Variation*	*n*	*p*
24 - 27	143.8 (± 7.7)	142.5 (± 7.7)	-1.38 (± 6.1)	61	0.082^1^
28 - 31	139.5 (± 7.7)	140.2 (± 8.1)	0.65 (± 7.2)	60	0.488^1^
32 - 35	139.1 (± 9.6)	139.2 (± 9.3)	0.02 (± 9.0)	64	0.989^1^
36 - 40	137.3 (± 8.9)	138.8 (± 11.2)	1.41 (±10.0)	58	0.285^1^
	*FM (mean ± SD)*				
			
24 - 27	16.2 (± 12.4)	19.1 (± 18.7)	2.9 (± 13.9)	61	0.240^2^
28 - 31	20.0 (± 15.6)	21.4 (± 18.5)	1.4 (± 20.2)	60	0.334^2^
32 - 35	22.9 (± 22.9)	21.8 (± 22.2)	-1.0 (± 23.3)	64	0.478^2^
36 - 40	21.4 (± 17.3)	23.6 (± 21.0)	2.2 (± 22.7)	58	0.636^2^
	*A (mean ± SD)*				
			
24 - 27	3.1 (± 2.6)	3.7 (± 3.0)	0.6 (± 2.4)	61	0.117^2^
28 - 31	4.6 (± 4.1)	4.9 (± 4.8)	0.4 (± 4.9)	60	0.983^2^
32 - 35	6.0 (± 5.4)	6.4 (± 4.6)	0.3 (± 5.6)	64	0.658^2^
36 - 40	7.7 (± 5.8)	8.3 (± 5.8)	0.6 (± 5.5)	58	0.402^2^
	*Variability of FHR (bpm ± SD)*				
			
24 - 27	8.5 (± 3.4)	10.1 (± 5.5)	1.6 (± 4.5)	61	**0.011**^2^
28 - 31	10.0 (± 5.0)	9.7 (± 4.6)	-0.3 (± 3.4)	60	0.930^2^
32 - 35	10.0 (± 5.0)	9.8 (± 5.3)	-0.2 (± 4.8)	64	0.701^2^
36 - 40	9.9 (± 4.7)	10.2 (± 4.8)	0.3 (± 4.8)	58	0.458^2^

^1 ^Student's t-test for related samples

^2 ^Wilcoxon's non-parametric test for related samples

Values ares expressed as means **± **Standard Deviation (SD).

**Figure 2:**
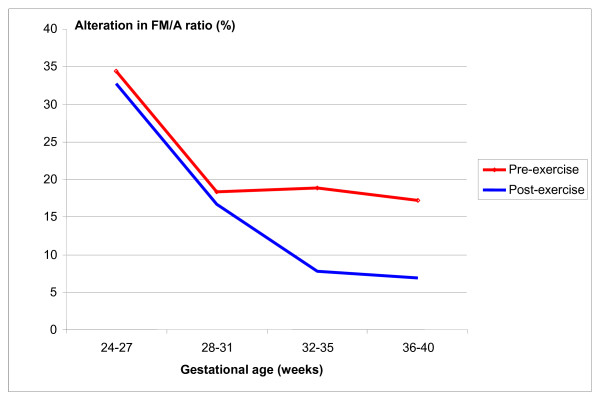
**Percentage of alteration in FM/A ratio prior to and following moderate physical exercise in water, according to gestational age**.

Exams showing a change in the ideal FM/A ratio (at least 2/2 in 20 minutes), as well as in spontaneous decelerations, were infrequent and did not differ significantly before and after water aerobics, as shown in Table 3 and Figure 2. With the exception of the 32-35 week gestational age-group in which no episode of deceleration was recorded, these episodes occurred physiologically both prior to and following the water aerobics sessions in around 2-6% of examinations.

**Table 3:** Proportion of alteration in the ratio between fetal movements and accelerations (FM/A) and the presence of decelerations recorded by cardiotocography (CTG) prior to and following moderate physical exercise in water, according to gestational age.

*Gestational age (weeks)*	*% altered FM/A*		*n*	*p *^1^
			
	*CTG before*	*CTG after*		
24 - 27	34.4	32.8	61	> 0.999
28 - 31	18.3	16.7	60	> 0.999
32 - 35	18.8	7.8	64	0.065
36 - 40	17.2	6.9	58	0.109
	***% with deceleration***			
			
24 - 27	4.9	6.6	61	> 0.999
28 - 31	1.7	3.3	60	> 0.999
32 - 35	-	-	64	--
36 - 40	5.2	5.2	58	> 0.999

^1 ^McNemar's test, binomial distribution

## Discussion

The findings of the present study show that, in general, there were no significant variations in cardiotocography parameters between the evaluations carried out prior to and immediately following sessions of water aerobics in pregnant women in the gestational age-groups evaluated. The only variable in which a statistically significant difference was found was variability of fetal heart rate, which was significantly greater following exercise only in the women with gestational age between 24 and 27 weeks. In addition, another important finding of this study was the presence of an alteration in the ratio between the number of fetal body movements and accelerations, which varied from 34% at 24-27 weeks to 7-17% at 36-40 weeks; however, no statistically significant differences were found in these parameters between pre- and post-exercise evaluations as well. Finally, spontaneous decelerations in FHR were detected in a small percentage of around 2-7% of cardiotocography exams in the majority of gestational age-groups. Again, no relationship was found with physical activity.

The absence of significant variations in basal FHR between the two time-periods is in agreement with data already published in the literature on studies in pregnant women carrying out physical activity in water [9,13,14]. This safety with respect to fetal heart rate appears to be a characteristic more specifically related to physical activity of moderate intensity practiced in water, since there appears to be a tendency for FHR to increase following land-based physical activity [15]. Nevertheless, even out of water, some studies also showed that physical exercise does not appear to significantly alter FHR [7,16,17]. The same would appear to be true for fetal body movements, no statistically significant differences having been found in the mean number of fetal body movements. According to the literature, fetal body movements have rarely been studied. One relatively old study refers to an increase in movements following moderate exercise [18] and another refers to a reduction in fetal body movements in the first five minutes after exercise performed on a bicycle [19]. These results could be indirectly related to the previous findings on neonatal safety reached with similar program of water aerobics for low risk pregnant women in a randomized controlled trial [20,21].

Although the number of accelerations did not vary significantly between measurements taken before and after physical exercise, there would appear to be a trend towards an increase in their number with an increase in gestational age. This parameter has not been evaluated systematically in the studies that have been published on pregnant women exercising in water; however, this trend has been reported previously in pregnant women carrying out physical exercise on bicycles [7]. Nevertheless, most of the studies evaluating the number of accelerations have reported little or no influence of the practice of physical activity by pregnant women on this variable [7,16,17,19], and rarely any reduction in the number of accelerations following exercise [22]. Anyway, we think that is worth to know that some low risk pregnant women can show an altered ratio between fetal body movements and accelerations with no pathological meaning.

The variability in FHR was the only parameter studied in which a significant increase was found between the pre- and post-exercise moments, exclusively in the group of women with the lowest gestational age. These results were rather unexpected. Although this variable has not yet been comprehensively studied in physical activity in water, studies using land-based exercise have shown an absence of association between variability in FHR and maternal physical activity [7], or even an increase in the proportion of cases in which a reduction was recorded following exercise [16,22].

The altered FM/A ratio, as well as spontaneous decelerations, were infrequent and did not differ significantly between pre-water aerobic measurements and post-exercise measurements. The association between fetal body movement and accelerations in FHR has yet to be better studied, especially in women with immersion in water. On the other hand, the finding of transitory fetal bradycardia (deceleration) has already been reported in various other studies in up to 20% of cases of pregnant women practicing physical activity [7,16,17,19,22], but has been reported more frequently in women exercising on a bicycle than swimming [9]. These results are important because they show that decelerations in FHR may be associated both with the practice of moderate physical exercise during pregnancy and with rest, and that this parameter has no pathological significance. It could also be associated as well with fetal activity status. Although these findings have been reproduced consistently, there is still little data available to healthcare professionals on these characteristics of FHR, and this may theoretically lead to the practice of iatrogenic obstetrical measures.

Some possible limitations of the present study may have restricted the scope of our results. First, the original study plan was to follow-up the same group of women throughout pregnancy to monitor FHR at different gestational ages and to evaluate the variation in the study parameters over time. Unfortunately, this was not possible because these women frequently failed to attend scheduled water aerobics sessions because of family or professional commitments. Therefore, a different sample of women had to be used for each gestational age-group, and we were, therefore, unable to carry out those planned evaluations. Other factors could possibly be pointed out, like the increase in the amniotic fluid volume after water aerobics [23] that could influence the number of fetal body movements, although this was not found; a possible reduction in maternal glucose levels resulting from energy spent during physical activity [24], which may have contributed towards altering FHR parameters following water aerobics. Confirmation of these possibilities would only have been possible if these women had been scanned with ultrasound and metabolically controlled during exercise, and this was not done.

Physical activity is currently considered desirable for the maintenance of physical and mental health. Studies carried out in low-risk pregnant women who exercise throughout pregnancy show benefits to both mother and fetus, since moderate intensity exercise, both land- and water-based, is safe, irrespective of whether the woman is sedentary or not. For these women, water-based exercise offers several advantages including fewer risks of joint lesions and a reduction in lower limb edema [25], as well as an increase in the amniotic fluid index [23] and an improvement in cardiovascular and respiratory adaptation, with no changes in fetal heart rate due to immersion [26]. Although more detailed studies on fetal physiology during maternal physical activity in water need to be carried out, the findings currently available, including the data resulting from the present study, suggest that this practice is safe, and that it should be recommended to pregnant women who are able and willing to carry it out.

## Competing interests

The authors report no conflict of interests. The authors alone are responsible for the content and writing of the paper.

## Authors' contributions

JGC had the original idea for the study. CS and BGP wrote the first version of the proposal. JGC got the grant for implementation of the study. CS and SRC were responsible for data collection. CS, BGP, RIP, SRC, and JGC were responsible for data analysis. CS and BGP wrote the first draft of the paper and then all the others gave important inputs and suggestions for interpretation and improvement of the manuscript. All authors have read the final version of the article and agreed with it.
